# Multi-Variant Pathway Association Analysis Reveals the Importance of Genetic Determinants of Estrogen Metabolism in Breast and Endometrial Cancer Susceptibility

**DOI:** 10.1371/journal.pgen.1001012

**Published:** 2010-07-01

**Authors:** Yen Ling Low, Yuqing Li, Keith Humphreys, Anbupalam Thalamuthu, Yi Li, Hatef Darabi, Sara Wedrén, Carine Bonnard, Kamila Czene, Mark M. Iles, Tuomas Heikkinen, Kristiina Aittomäki, Carl Blomqvist, Heli Nevanlinna, Per Hall, Edison T. Liu, Jianjun Liu

**Affiliations:** 1 Human Genetics, Genome Institute of Singapore, Singapore, Singapore; 2 Department of Medical Epidemiology and Biostatistics, Karolinska Institutet, Stockholm, Sweden; 3 Institute for Environmental Medicine, Karolinska Institutet, Stockholm, Sweden; 4 Section of Epidemiology and Biostatistics, Leeds Institute of Molecular Medicine, St. James's University Hospital, Leeds, United Kingdom; 5 Department of Obstetrics and Gynecology, Helsinki University Central Hospital, Helsinki, Finland; 6 Department of Clinical Genetics, Helsinki University Central Hospital, Helsinki, Finland; 7 Department of Oncology, Helsinki University Central Hospital, Helsinki, Finland; 8 Department of Oncology, Radiology, and Clinical Immunology, Uppsala University Hospital, Uppsala, Sweden; 9 Cancer Biology, Genome Institute of Singapore, Singapore, Singapore; University of Washington, United States of America

## Abstract

Despite the central role of estrogen exposure in breast and endometrial cancer development and numerous studies of genes in the estrogen metabolic pathway, polymorphisms within the pathway have not been consistently associated with these cancers. We posit that this is due to the complexity of multiple weak genetic effects within the metabolic pathway that can only be effectively detected through multi-variant analysis. We conducted a comprehensive association analysis of the estrogen metabolic pathway by interrogating 239 tagSNPs within 35 genes of the pathway in three tumor samples. The discovery sample consisted of 1,596 breast cancer cases, 719 endometrial cancer cases, and 1,730 controls from Sweden; and the validation sample included 2,245 breast cancer cases and 1,287 controls from Finland. We performed admixture maximum likelihood (AML)–based global tests to evaluate the cumulative effect from multiple SNPs within the whole metabolic pathway and three sub-pathways for androgen synthesis, androgen-to-estrogen conversion, and estrogen removal. In the discovery sample, although no single polymorphism was significant after correction for multiple testing, the pathway-based AML global test suggested association with both breast (*p*_global_ = 0.034) and endometrial (*p*_global_ = 0.052) cancers. Further testing revealed the association to be focused on polymorphisms within the androgen-to-estrogen conversion sub-pathway, for both breast (*p*_global_ = 0.008) and endometrial cancer (*p*_global_ = 0.014). The sub-pathway association was validated in the Finnish sample of breast cancer (*p*_global_ = 0.015). Further tumor subtype analysis demonstrated that the association of the androgen-to-estrogen conversion sub-pathway was confined to postmenopausal women with sporadic estrogen receptor positive tumors (*p*_global_ = 0.0003). Gene-based AML analysis suggested *CYP19A1* and *UGT2B4* to be the major players within the sub-pathway. Our study indicates that the composite genetic determinants related to the androgen–estrogen conversion are important for the induction of two hormone-associated cancers, particularly for the hormone-driven breast tumour subtypes.

## Author Summary

Estrogen exposure is the most important risk factor for breast and endometrial cancers. Genetic variation of the genes involved in estrogen metabolism has, however, not been consistently associated with these two cancers. We posited that the genetic risk associated with the estrogen metabolic genes is likely to be carried by multiple variants and is therefore most effectively detected by multi-variant analysis. We carried out a comprehensive association analysis of the estrogen metabolic pathway by interrogating SNPs within 35 genes of the pathway in three tumor samples from Sweden and Finland. Through pathway-based multi-variant association analysis, we showed that the genetic variation within the estrogen metabolic pathway is associated with risk for breast and endometrial cancers and that the genetic variation within the genes involved in androgen-to-estrogen conversion is particularly important for the development of ER–positive and sporadic breast tumors in postmenopausal women. Our study has demonstrated that the influence of genetic variation on hormone exposure has an impact on breast cancer development, especially on the development of hormone-driven breast tumor subtypes. Our study has also highlighted that future genetic studies of the estrogen metabolic genes should focus on the androgen-to-estrogen conversion process.

## Introduction

Estrogen exposure is critical for the development of both breast and endometrial cancers and represents the most well-established risk factors for both diseases. Estrogen is a metabolic product whose circulating level is determined by de novo synthesis, conversion from other steroid hormones, and mechanisms of estrogen elimination. These metabolic processes are regulated by a network of enzymes encoded by different genes, suggesting that genetic variation within these metabolic genes may impact on breast and endometrial cancer risk. Genetic variation within the estrogen metabolic pathway has been intensively investigated, mostly by analyzing single variant effects in a limited number of candidate genes, SNPs and study subjects. The inadequacies of study design and analytical methodology have caused these studies to be underpowered for detecting moderate genetic effects which, not surprisingly, has led to inconsistent results [1]–[8]. We surmised that strategies for assessing the synergistic effect of multiple genetic variants within the estrogen metabolic pathway may provide a more realistic determination of genetic effect than a single gene, single SNP approach.

Herein, we present a comprehensive analysis of genetic variation in the estrogen metabolism pathway and its association with breast and endometrial cancer risk using a pathway-based approach.

## Results

### Single SNP Association Analysis

We performed single SNP association analysis in 1596 breast cancer cases, 719 endometrial cancer cases and 1730 population controls from Sweden. Of the 239 tagSNPs analyzed, 17 SNPs (7.1%) had p-values less than 0.05 for breast cancer, and 18 SNPs (7.5%) had p-values less than 0.05 for endometrial cancer (Table S4 and Table S5). For breast cancer, the smallest p-value was 0.00034 at rs7167936 within *CYP19A1*, and for endometrial cancer, the smallest p-value was 0.00017 at rs12595627 in *CYP19A1*. The single-SNP associations were all moderate. Only rs12595627 (for endometrial cancer) survived the conservative Bonferroni correction for multiple testing at α = 0.05. Overall, however, the single-SNP p values appeared to deviate from their null distribution of no association (formally tested below). The single-SNP associations were suggestive, but instead of any single variant having a strong effect, there appeared to be multiple weak associations within the metabolic pathway.

### Multi-SNP Pathway Analysis

**Figure 1:**
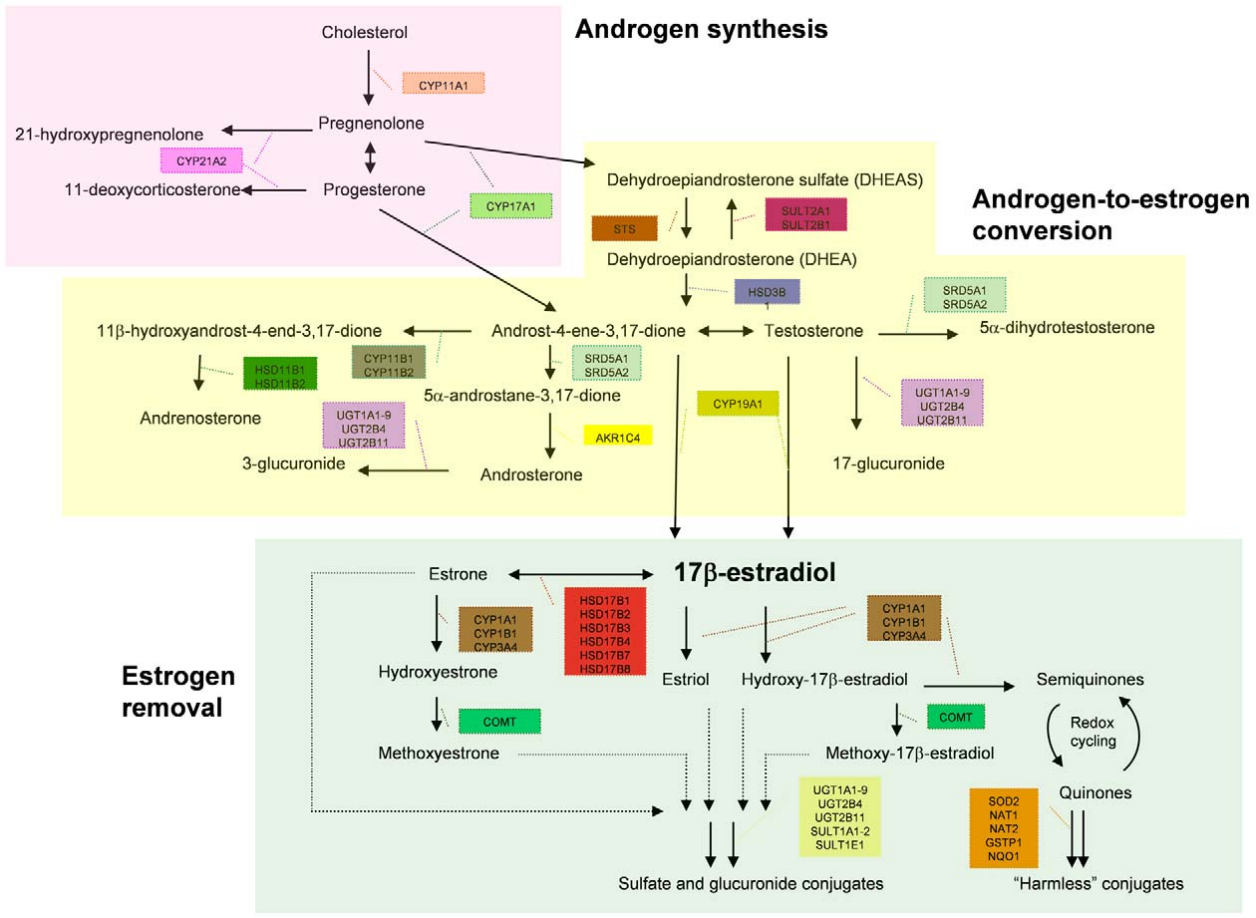
Subdivision of the estrogen metabolic pathway. This diagram shows how the 35 metabolic genes analysed in this study are involved in different steps of the estrogen metabolism. It further shows how the genes are divided into the three groups involved in androgen synthesis, estrogen synthesis and estrogen removal for sub-pathway-based association analysis.

To evaluate the cumulative effect from multiple variants we employed the AML method [9] that assesses the experiment-wide significance of association by analyzing multiple SNPs through a single global test. The whole metabolic pathway can be sub-divided into three *a priori* defined sub-pathways, each performing specific metabolic function (Figure 1). Sub-pathway 1 is involved in the synthesis of androgen, sub-pathway 2 is involved in the conversion of androgens to estrogens, and sub-pathway 3 is responsible for removing estrogens. To investigate whether there is multi-SNP association for the whole pathway and whether any of the three sub-pathways is particularly important in influencing disease risk, we performed the progressive pathway-based global test on the whole metabolic pathway as well as the three sub-pathways using the AML method. The global test yielded marginally significant association for the whole metabolic pathway in both breast (*p*_global_ = 0.034) and endometrial (*p*_global_ = 0.052) cancers (Table 1). Dividing the metabolic pathway into three functional sub-pathways for the global test revealed strong association between the androgen-to-estrogen conversion sub-pathway and both breast (*p*_global_ = 0.008) and endometrial (*p*_global_ = 0.014) cancer (Table 1). The association evidence survived correction for performing 4 pathway-based tests in each cancer (*p*_global corrected_ = 0.032 for breast and 0.056 for endometrial). In contrast, the other two sub-pathways showed no association with either form of cancer. For approximately half of the Swedish subjects in the breast cancer study (797 cases and 764 controls) we have genome wide association study (GWAS) data available. We used this to assess the possible influence of population stratification on our results. For the GWAS dataset, the genomic inflation factor, λ_gc_, was1.015. Assuming an equal level of population stratification (in terms of the fixation index F_ST_) in the current study and the GWAS sub-study, we estimated the genomic inflation factor, λ_gc_, to be 1.030 in the current study, using the relationship between F_ST_, sample size and λ_gc_ described in [10]. Using the λ_gc_ value of 1.030 for genomic control-based correction of population stratification, the corrected global AML p-values for breast cancer are 0.052 for the entire pathway and 0.011 for the androgen-estrogen conversion sub-pathway, leaving our results largely unchanged. Even if λ_gc_ was as large as 1.05 in the current study, the global test p-value for the androgen-estrogen conversion sub-pathway would still be as low as 0.014. To further ensure that the observed associations could not be due to the employment of 319 paraffin-embedded tissue samples in the analysis, we re-ran analyses excluding 319 paraffin-embedded tissue samples, and (at the same time) excluding 33 SNPs with call rates of less than 95%. Results were very similar. For example, for breast cancer, p-values were 0.028 and 0.009 for the entire pathway and for the androgen-estrogen conversion sub-pathway, respectively. To validate the association in the androgen-to-estrogen conversion sub-pathway, we genotyped the 120 SNPs of this sub-pathway in an additional 2245 breast cancer cases and 1287 controls from Finland and performed the same AML analysis by using the 118 successfully genotyped SNPs. The validation analysis in the Finnish sample revealed similar evidence of association between the androgen-to-estrogen conversion sub-pathway and breast cancer (*p*_global_ = 0.015) (Table 1). The non-centrality parameter from the AML analysis of the androgen-to-estrogen conversion sub-pathway, which represents the size of the common effect of the associated SNPs, was estimated as 2.90 for the Swedish sample and 2.94 for the Finnish sample. The similar values indicate a consistent size of the genetic effect in the two samples. A joint analysis of the Swedish and Finnish samples further yielded a global p-value of 0.001 (Table 1). The SNPs with the lowest p-values in the Finnish sample are listed in Table S6.

**Table 1:** P values of the global tests of genetic association between the SNPs in the estrogen metabolic pathways and breast/endometrial cancer risk.

	Swedish		Finnish	Swedish and Finnish
	Breast Cancer	Endometrial Cancer	Breast Cancer	Breast Cancer
Whole Pathway (239 SNPs)	0.034	0.052	–	
Androgen Synthesis (11 SNPs)	0.397	0.381	–	
Androgen-Estrogen Conversion (120 SNPs)*	0.008	0.014	0.015	0.001
Estrogen Removal (144 SNPs)*	0.172	0.385	–	

P-values were based on 2500 permutations.

*:36 SNPs are overlapped.

### Analysis of the Androgen-to-Estrogen Conversion Sub-Pathway in Breast Cancer Patient Subgroups

Hormone-related risk factors may play a differential role in breast cancer subtypes. In particular, estrogens appear to drive the development of ER positive tumors. This prompted us to investigate the association in the androgen-to-estrogen conversion sub-pathway in hormone-related breast tumor subtypes. As surrogate markers for hormone driven tumour subtypes we constructed variables as combinations of menopausal status, family history and estrogen receptor (ER) status and divided all the patients into subgroups. We then compared subgroups of patients, defined on values of these variables, with controls, to evaluate the role of the androgen-to-estrogen conversion sub-pathway in different patient subgroups. First, we compared patient subgroups against all the controls in the combined Swedish and Finnish samples. The subgroup results showed that in the combined samples, significant association was observed in postmenopausal patients (*p*_global_ = 0.009 and 0.018 respectively), postmenopausal patients without family history (*p*_global_ = 0.001 and 0.04 respectively), and postmenopausal patients with estrogen receptor positive (ER+) tumors (*p*_global_ = 0.0006 and 0.05 respectively) (Table 2). No significant association was observed in either premenopausal patients or postmenopausal patients with family history or estrogen receptor negative (ER−) tumors.

**Table 2:** Patient subgroup analysis of the androgen-to-estrogen conversion sub-pathway.

		All Cases	Menopausal Status		Family History		ER Status	
			Pre	Post (PM)	PM Familial	PM Sporadic (PMS)	PMS ER+	PMS ER−
Swedish Sample	# controls	1518						
	# cases	1555	–	1545	244	1260	661	183
	P_global_	0.008	–	0.009	0.23	0.001	0.0006	0.65
Finnish Sample	# controls	1287						
	# cases	2245	498	1176	313	853	704	137
	P_global_	0.015	0.10	0.018	0.43	0.040	0.050	0.36
Joint AML Analysis	# controls	2805						
	# cases	3800	498	2721	557	2113	1365	320
	P_global_	0.001	0.10	0.002	0.33	0.0005	0.0003	0.57

All the P_global_ values are based on 5,000 permutations and reflect comparisons of various patient subgroups with all the controls.

Then, to rule out the possibility that the above subgroup results were caused by the mismatch between the patient subgroups and the controls in terms of the variables which defined patient subgroups, we performed the second subgroup analysis where the controls were also divided into subgroups according to family history and menopausal status (Table 3). The second subgroup analysis was only performed in the Swedish sample, because the Finnish controls lack information on family history and menopausal status. This yielded similar evidence for the association of the sub-pathway with the hormone-driven subtypes of breast cancer as in Table 2.

**Table 3:** Subgroup analysis of the androgen-to-estrogen conversion sub-pathway in the Swedish samples.

	All Cases	Menopausal Status		Family History		ER Status	
		Pre	Post (PM)	PM Familial	PM Sporadic(PMS)	PMS ER+	PMS ER−
# controls	1518		1505	128	1253	1253	1253
# cases	1555	–	1545	244	1260	661	183
P_global_	0.008	–	0.009	0.896	0.002	0.001	0.618

P-values for tests using PMS ER+ and PMS ER− patient sub-groups are based on comparisons with 1253 PMS controls.

### Analysis of Reproductive Risk Factors' Impact on the Association of the Androgen-to-Estrogen Conversion Sub-Pathway with Breast Cancer

We further investigated the impact of reproductive risk factors on the genetic association of the androgen-to-estrogen conversion sub-pathway with breast cancer. Because the risk factor information is not available for the Finnish controls, the analysis of the reproductive risk factors was performed in the Swedish samples where information on such factors is available. We performed the AML analysis of the androgen-to-estrogen conversion sub-pathway with adjustment for the reproductive risk factors (parity, age at the first birth, age at menarche and age of menopause) and HRT use. We investigated this primarily to assess whether any of the reproductive risk factors could be in the causal pathway. Since p-values remained almost unchanged in all analyses (Table 4), it appears that none of the reproductive risk factors are likely to be in the causal pathway.

**Table 4:** P_global_ values for the androgen-to-estrogen sub-pathway for all cases and for PMS ER+ cases in the Swedish sample set, adjusted for reproductive and hormone risk factors.

Adjusted Reproductive Variables	All cases		PMS ER+	
	Sample Size (case/control)	P_global_	Sample Size (case/control)	P_global_
Unadjusted	1555/1518	0.008	661/1518	0.0006
HRT use*	1541/1493	0.005	651/1493	0.0008
Parity*	1555/1518	0.0088	661/1518	0.0014
Age at first birth*	1323/1370	0.0176	563/1370	0.0016
Age at menarche*	1411/1390	0.0036	595/1390	0.0004
Age at menopause*	1545/1505	0.0102	658/1505	0.0016

*: HRT use, the AML Pglobal values were adjusted by a categorical variable, HRT/nonHRT. Similarly, Parity, adjusted by none/one or more children; Age at first time birth, adjusted by <25yrs, 25–30yrs, 30–35yrs and > = 35yrs; Age at menarche, adjusted by > = 14yrs, 12–13yrs and < = 12yrs; Age at menopause, adjusted by <45yrs, 45–50yrs, 50–55yrs and > = 55yrs. All AML Pglobal values are based on 5,000 permutations. PMS: postmenopausal and sporadic.

### Gene-Based Analysis of the Androgen-to-Estrogen Conversion Sub-Pathway in Breast and Endometrial Cancers

Attempting to refine the association within the androgen-to-estrogen conversion sub-pathway, we performed a gene-based AML analysis in the combined Swedish/Finnish breast cancer sample and the Swedish endometrial cancer sample. Among the 15 genes tested (Table 5), strong association was observed for *CYP19A1* with both breast (*p*_global_ = 0.003) and endometrial (*p*_global_ = 0.006) cancer and *UGT2B4* (*p*_global_ = 0.002) with breast cancer only. The associations in breast cancer survived correction for multiple testing of 15 genes (*p*_global corrected_ = 0.045 for *CYP19A1* and 0.03 for *UGT2B4*). We also observed suggestive association for *UGT2B11* in breast and endometrial cancer as well as for *HSD11B1*, *SULT2A1* and *SULT2B1* in breast cancer. Consistent with the pathway-based associations, the gene-based associations are generally more significant in sporadic postmenopausal patient samples than in the whole breast cancer sample (except *SULT2B1*). Furthermore, the importance of *CYP19A1* and *UGT2B4* in breast cancer risk is supported by the fact that excluding either gene from the global test of the sub-pathway reduced the global significance of association for the sub-pathway, from 0.0015 to 0.011 for *CYP19A1*, and to 0.010 for *UGT2B4*. However, the fact that the association for the sub-pathway remained significant, after excluding either gene, suggests that, although *CYP19A1* and *UGT2B4* are the major players, genetic variation within other genes also contributes to the association within the sub-pathway.

**Table 5:** Gene-based AML P_global_ values for the 15 genes within the androgen-to-estrogen conversion sub-pathway.

Genes	# SNPs	Breast Cancer *			Endometrial
		All Cases	PMS	PMS ER+	Cancer
AKR1C4	11	0.121	0.098	0.113	0.729
CYP11B1	2	0.595	0.692	0.619	0.663
CYP11B2	4	0.390	0.496	0.665	0.863
CYP19A1	15	0.006	0.003	0.013	0.006
HSD11B1	9	0.181	0.125	0.026	0.701
HSD11B2	6	0.130	0.244	0.096	0.778
HSD3B1	7	0.549	0.551	0.108	0.065
SRD5A1	5	0.870	0.852	0.851	0.325
SRD5A2	7	0.267	0.151	0.190	0.265
STS	9	0.393	0.582	0.997	0.806
SULT2A1	8	0.332	0.040	0.080	0.535
SULT2B1	12	0.028	0.190	0.193	0.784
UGT1A1-9	12	0.378	0.413	0.205	0.888
UGT2B11	7	0.179	0.078	0.027	0.047
UGT2B4	7	0.002	0.003	0.003	0.31

*:PMS, Postmenopausal Sporadic Cases; PMS ER+, Postmenopausal Sporadic Cases with ER+ tumors; the AML P_global_ values for breast cancer were based on both the Swedish and Finnish samples and calculated using Fisher's method. All AML P_global_ values are based on 5,000 permutations.

## Discussion

Our pathway-based multi-SNP association analysis revealed a significant association between genetic variants in the androgen-to-estrogen conversion sub-pathway and the risk of two hormone dependent cancers. The association was particularly strong for ER+, sporadic breast cancer. Single SNP analysis did not reveal a similar association. We used the AML-based multi-SNP analysis, which has been shown to be more powerful than single SNP tests to yield significant and consistent association, when genetic risk is carried by multiple risk alleles each with moderate effect [11].

Pathway-based approaches are just beginning to be applied in association analysis [12]. Recently, an association study of 9 candidate gene groups (involving 120 candidate genes) was performed in breast cancer by using the AML approach, and interestingly, only the group of 8 genes involved in the steroid hormone signalling were significantly associated [13]. Our study has moved one step further and highlights the fact that the power of the pathway-based association analysis can be increased when analysis is guided by well-defined biological information. We believe that pathway approaches have potential to move genome-wide association studies beyond their initial success of identifying some ‘low-hanging fruits’ to revealing many weak genetic risk alleles that have been missed by single SNP analysis.

Unless one enzyme is the rate limiting step for the entire metabolic pathway, it is not likely that small functional perturbations of individual variants would have a major impact on the overall effect of the metabolic pathway. To test the hypothesis that several genetic variants, each conferring weak to moderate effects, contribute to genetic risk, we adopted a systematic pathway-based approach for association analysis by testing the joint effect of multiple genetic variants in a progressive fashion from the whole metabolic pathway to biochemical sub-pathways and further down to individual genes. Such a progressive approach allows us to not only establish consistent association in three cancer samples from two different populations but also to refine the association of the androgen-to-estrogen conversion component of the metabolic pathway. Our study may therefore have advanced our understanding of the role of estrongen metabolism in breast and endometrial cancers by 1) accounting for the ambiguity surrounding the genetic association results and 2) indicating the androgen-to-estrogen conversion to be the important component of the metabolic pathway in modulating the risk and therefore to be a worthy focus for future studies.

After menopause, ovarian estrogen production dramatically declines and conversion of adrenal androgens to estrogens in peripheral tissues becomes the major source of circulating estrogens. The final step of this conversion is catalyzed by aromatase, encoded by *CYP19A1* [11]. Thus, there is biological plausibility in the association between *CYP19A1* polymorphisms and postmenopausal breast cancer. Moreover, pharmacological inhibition of aromatase prevents recurrences in postmenopausal women with estrogen-receptor-positive breast cancer and new contralateral primaries [14], which has challenged the previous routine of a 5-year course of tamoxifen alone [15]. Our study has advanced our understanding of *CYP19A1* by suggesting that the modulation of aromatase activity by either germ-line variation or pharmacological agents can influence the development of ER+ tumour in postmenopausal women. Furthermore, the convergence of genetic and pharmacological effects of *CYP19A1* also raises therapeutic possibilities. For example, other genes implicated by our genetic study, such as *UGT2B4*, might also be pharmacological targets for treating breast cancer.

Hormone exposure is a common risk factor for breast and endometrial cancer. Our employment of the three samples of two different hormone-related cancers from two different populations allowed us to apply a very stringent criterion for declaring an association. Furthermore, results of our breast cancer patient subgroup analysis indicate that the genetic determinants within the androgen-to-estrogen conversion sub-pathway may play a more prominent role in postmenopausal women with sporadic ER+ tumors, further suggesting that the modulation of hormone exposure by genetic variation may have a differential impact on breast tumor subtypes. Endogenous sex hormone level appears to be associated with breast cancer risk in postmenopausal women [16], and particularly with the risk of ER+/PR+ breast tumors [17]. The effect of hormone-related factors on breast cancer risk apparently differs by ER status [18] and menopause status [19], [20]. It could also differ by the status of family history of the disease, as suggested by a recent study showing that most cases of hereditary breast cancer are probably not related to cumulative hormone exposure [21]. Our findings may have therefore advanced the development of a general model for breast cancer risk: hormonal factors, both genetic and reproductive, can play a key role in the genesis of post-menopausal and “sporadic” breast cancer, whereas genes involved in DNA repair, checkpoints, and genetic stability (such as *BRCA1*, *BRCA2*, *p53*, *ATM*, *CHK2*) appear to be more involved in predominantly breast cancers associated with family history of disease.

It is worth noting that the contribution of genetic polymorphisms to risk is a function of both their prevalence and penetrance and thus the relative importance of individual SNPs may vary from population to population. More studies in different populations are needed to fully understand the role of the androgen-to-estrogen conversion sub-pathway in breast cancer. We also want to highlight that our results are of genetic association in nature, and further studies are needed to confirm the findings and to identify functional variants causally linked to cancer risk.

## Materials and Methods

### Study Subjects

Swedish subjects were from a population-based case control study of breast and endometrial cancer as described [22], [23]. Briefly, the study included all incident primary invasive breast and endometrial cancers among Swedish-born postmenopausal women between 50 and 74 years of age at diagnosis, diagnosed with breast cancer between October 1993 and March 1995 and endometrial cancer between January 1994 and December 1995. All cases were identified through six regional cancer registries in Sweden, and all controls were randomly selected from the Swedish Registry of Total Population and frequency matched to the expected age distribution of the cases.

Finnish breast cancer cases consist of two series of unselected breast cancer patients and additional familial cases ascertained at the Helsinki University Central Hospital. The first series of 884 patients was collected in 1997–1998 and 2000 and covers 79% of all consecutive, newly diagnosed cases during the collection periods [24], [25]. The second series, containing 986 consecutive newly diagnosed patients, was collected in 2001–2004 and covers 87% of all such patients treated at the hospital during the collection period [26]. An additional 538 familial breast cancer cases were collected at the same hospital as described [27]–[30]. 1287 anonymous, healthy female population controls were collected from the same geographical regions in Southern Finland as the cases and have been used in several studies previously [31]–[33].

Risk factor information and tumour characteristics were available for all the Swedish samples and the Finnish cases, but were missing for the Finnish controls. The Finnish samples (mean age = 56 for the cases and 41 for the controls) were younger than the Swedish samples (mean age = 63 for both the cases and controls). All the risk factor and tumour characteristics information of the subjects are summarized in Table S1 and Table S2.

Written informed consent was obtained from all participating subjects, and the study was approved by the Institutional Review Boards in Sweden, Finland and at the National University of Singapore.

### DNA Isolation

DNA was extracted from 4 ml of whole blood using the QIAamp DNA Blood Maxi Kit (Qiagen)and non-malignant cells in paraffin-embedded tissue using a standard phenol/chloroform/isoamyl alcohol protocol [34].

### Gene and SNP Selection

We selected 35 genes involved in estradiol or estrone metabolism and expressed in the breast (based on published literatures). We selected 1007 single nucleotide polymorphisms (SNPs) in these genes and their 30kb flanking sequences from the dbSNP (build 124) and Celera databases, aiming for a marker density of at least one SNP per 5kb (Table S3). These SNPs were genotyped in 92 Swedish control samples to assess linkage disequilibrium pattern and coverage. Haplotypes were reconstructed using the PLEM algorithm [35] implemented in the *tagSNPs* program [36]. A subset of SNPs, tagSNPs, were selected based on the *R^2^* coefficient, which quantifies how well the tagSNP haplotypes predict the genotype or haplotypes an individual carries. We chose tagSNPs so that common SNP genotypes and haplotypes (frequency ≥0.03) were predicted with *R^2^*≥0.8 [37]. To evaluate our tagSNPs' performance in capturing unobserved SNPs within the genes, we performed a SNP-dropping analysis [38], [39]. In brief, each of the genotyped SNPs was dropped in turn and tagSNPs were selected from the remaining SNPs so that their haplotypes predicted the remaining SNPs with an *R^2^* value of 0.85. We then estimated how well the tagSNP haplotypes of the remaining SNPs predicted the dropped SNP, an evaluation that can provide an unbiased and accurate estimate of tagSNP performance [38], [39]. Overall, we selected and genotyped 302 tagSNPs from the 35 genes in all the Swedish cases and controls.

### Genotyping

Genotyping was performed using the Sequenom system (San Diego, California). All genotyping results were generated with positive and negative controls and checked by laboratory staff unaware of case-control status. Of the 302 tagSNPs, 42 SNPs failed in the development stage of Sequenom genotyping assays. SNPs with a call rate <85% (8 SNPs), minor allele frequency <1% (9 SNPs) or out of Hardy-Weinberg Equilibrium (p<0.05/252, 4 SNPs) were excluded from further analysis. Overall, 239 tagSNPs from the 35 genes were successfully genotyped (Table S3). The genotype concordance was >99%, suggesting high genotyping accuracy.

### Statistical Analysis

The Cochran-Armitage trend test was performed for each of the 239 SNPs. One approach for assessing the departure of the distribution of the (Cochran-Armitage) test statistics from the (global) null distribution (no SNPs associated) has been described by Tyrer et. al. [9]. The approach is based upon fitting a mixture model to the distribution of the test statistics, with two components, one representing SNPs which are independent of the case-control status, the other representing SNPs associated with case-control status. The Cochran-Armitage test statistics for the associated SNPs are assumed to all have the same (chi-squared) non-centrality parameter value. The distributed software for the “admixture maximum likelihood” (AML) test of Tyrer et. al. [9] calculates empirical p-values based on a “pseudo-likelihood ratio” test, comparing the ratio of values of the optimized likelihoods under the null and alternative hypotheses for the observed data, with the corresponding values obtained from a large number of data sets with case-control status permuted randomly. It also provides an estimate of the non-centrality parameter which is a measure of the common effect size of the associated SNPs within the pathway. We performed the AML-based global test of association for the full metabolic pathway as well as for 3 sub-pathways (see results section). In addition, we performed gene-specific analyses, using the AML-based global test on SNPs within genes, within the androgen-estrogen conversion sub-pathway. We also carried out AML tests adjusted for a non-genetic risk factor using software provided by the authors of Tyrer et al. [9].

## Supporting Information

Table S1 Selected characteristics of the subjects of the Swedish breast cancer sample.
(0.03 MB DOC)

Table S2 Tumour characteristics of the cases of Swedish and Finnish breast cancer samples.
(0.03 MB DOC)

Table S3 Genes and number of SNPs used in analyses.
(0.08 MB DOC)

Table S4 Twenty-five most significant SNPs for breast cancer in Swedish sample.
(0.07 MB DOC)

Table S5 Twenty-five most significant SNPs for endometrial cancer in Swedish sample.
(0.07 MB DOC)

Table S6 Twenty-five most significant SNPs for breast cancer in Finnish sample.
(0.07 MB DOC)
