# Supplementary material for: Multi-Variant Pathway Association Analysis Reveals the Importance of Genetic Determinants of Estrogen Metabolism in Breast and Endometrial Cancer Susceptibility
Source: PLoS Genet. 2010 Jul 1;6(7):e1001012. doi: 10.1371/journal.pgen.1001012 (PMC2895650; doi:10.1371/journal.pgen.1001012)
Supplement: Table S1 — Selected characteristics of the subjects of the Swedish breast cancer sample. (0.03 MB DOC) [file pgen.1001012.s001.doc]

**Table S1.** Selected characteristics of the subjects of the Swedish breast cancer sample

| **Characteristic** | **Number (all cases/controls)** | **All cases/controls** | **P value (2 tails)** |
| --- | --- | --- | --- |
|  |  | Mean |  |
| Age (years) | 1555/1518 | 63.34 / 63.14 | 0.3997 |
| Parity | 1555/1518 | 1.84 / 2.16 | <0.00001 |
| Age at first birth(years) | 1323/1370 | 25.37 / 24.73 | 0.0005 |
| Age at menarche (years) | 1411/1390 | 13.50 / 13.53 | 0.69 |
| Age at menopause (years) | 1545/1505 | 50.4 / 50.01 | 0.004 |
| Recent BMI (kg/m^2^) | 1546/1497 | 25.78 / 25.53 | 0.1074 |
|  |  | Percent |  |
| HRT use (yes/no) | 1541/1493 | 32.97 / 27.24 | 0.0006 |
| Family history (yes/no) | 1518/1381 | 16.21 / 9.27 | <0.00001 |
