# Supplementary material for: Multi-Variant Pathway Association Analysis Reveals the Importance of Genetic Determinants of Estrogen Metabolism in Breast and Endometrial Cancer Susceptibility
Source: PLoS Genet. 2010 Jul 1;6(7):e1001012. doi: 10.1371/journal.pgen.1001012 (PMC2895650; doi:10.1371/journal.pgen.1001012)
Supplement: Table S2 — Tumour characteristics of the cases of Swedish and Finnish breast cancer samples. (0.03 MB DOC) [file pgen.1001012.s002.doc]

**Table S2.** Tumour characteristics of the cases of Swedish and Finnish breast cancer samples

|  | **Swedish** | **Finnish** |
| --- | --- | --- |
| **Phenotype** | **Number of cases (Percent)** | **Number of cases (Percent)** |
| ER positive | 801 (78.6%) | 1718 (80.8%) |
| ER negative | 218 (21.4%) | 408 (19.2%) |
| Postmenopausal | 1545 (99%) | 1176 (70.3%) |
| Premenopausal | – | 498 (29.8%) |
| Sporadic | 1272 ( 83.8%) | 1302 (59.3%) |
| Familial | 246 (16.2%) | 895 (40.7%) |
