# Supplementary material for: Multi-Variant Pathway Association Analysis Reveals the Importance of Genetic Determinants of Estrogen Metabolism in Breast and Endometrial Cancer Susceptibility
Source: PLoS Genet. 2010 Jul 1;6(7):e1001012. doi: 10.1371/journal.pgen.1001012 (PMC2895650; doi:10.1371/journal.pgen.1001012)
Supplement: Table S3 — Genes and number of SNPs used in analyses. (0.08 MB DOC) [file pgen.1001012.s003.doc]

**Table S3. Genes and number of SNPs used in analyses**

| **Gene name** | **Chr** | **Start Position*** | **End Position*** | **Number of SNPs used in LD analysis** | **Number of tagSNPs used in association analysis** | **Coverage evaluation (r^2^>0.80)** |
| --- | --- | --- | --- | --- | --- | --- |
| AKR1C4 | 10 | 5225926 | 5252412 | 33 | 11 | 0.83 |
| COMT | 22 | 18307834 | 18338950 | 58 | 10 | 0.74 |
| CYP11A1 | 15 | 72415693 | 72448634 | 31 | 6 | 0.90 |
| CYP11B1 | 8 | 143949280 | 143959738 | 6 | 2 | 0.4 |
| CYP11B2 | 8 | 143987483 | 143997761 | 9 | 4 | 0.78 |
| CYP17A1 | 10 | 104578782 | 104588780 | 8 | 5 | 0.75 |
| CYP19A1 | 15 | 49286046 | 49419599 | 53 | 14 | 0.93 |
| CYP1A1-2 | 15 | 72797437 | 72837494 | 12 | 5 | 0.37 |
| CYP1B1 | 2 | 38146750 | 38158296 | 9 | 5 | 0.83 |
| CYP21A2 | 6 | 32114061 | 32117398 | 4 | 0 | - |
| CYP3A4-5 | 7 | 99082259 | 99221244 | 18 | 6 | 0.98 |
| GSTP1 | 11 | 67106142 | 67112207 | 22 | 1 | - |
| HSD11B1 | 1 | 207924633 | 207976418 | 57 | 9 | 0.95 |
| HSD11B2 | 16 | 66021037 | 66030453 | 13 | 6 | 0.5 |
| HSD17B1 | 17 | 37953258 | 37962250 | 23 | 6 | 0.82 |
| HSD17B2 | 16 | 80624864 | 80691138 | 38 | 11 | 0.91 |
| HSD17B3 | 9 | 98035910 | 98105755 | 64 | 8 | 0.88 |
| HSD17B4 | 5 | 118814603 | 118907426 | 38 | 8 | 0.63 |
| HSD17B7 | 1 | 161027120 | 161049231 | 11 | 5 | 0.82 |
| HSD17B8 | 6 | 33280397 | 33282585 | 13 | 4 | 0.33 |
| HSD3B1 | 1 | 119849849 | 119860704 | 52 | 7 | 0.91 |
| NAT1 | 8 | 18111895 | 18125099 | 16 | 7 | 0.8 |
| NAT2 | 8 | 18293035 | 18303003 | 14 | 7 | 1 |
| NQO1 | 16 | 68299308 | 68319534 | 19 | 7 | 0.6 |
| SOD2 | 6 | 160018641 | 160035843 | 10 | 5 | 0.83 |
| SRD5A1 | 5 | 6685000 | 6724173 | 13 | 5 | 0.69 |
| SRD5A2 | 2 | 31601660 | 31660973 | 30 | 7 | 0.88 |
| STE (SULT1E1) | 4 | 70740020 | 70761959 | 12 | 7 | 0.88 |
| STS | X | 7145997 | 7284180 | 45 | 9 | 0.92 |
| SULT1A1-2 | 16 | 28509267 | 28543875 | 12 | 6 | 0.47 |
| SULT2A1 | 19 | 53064182 | 53082905 | 24 | 8 | 0.95 |
| SULT2B1 | 19 | 53745741 | 53795995 | 39 | 12 | 0.97 |
| UGT1A1-9 | 2 | 234189593 | 234348188 | 84 | 12 | 0.99 |
| UGT2B11 | 4 | 70100636 | 70115038 | 93 | 7 | 0.83 |
| UGT2B4 | 4 | 70378974 | 70397712 | 24 | 7 | 0.89 |
|  |  |  | Total | 1007 | 239 |  |

*:Based on the Mar 2006 human reference sequence (NCBI Build 36).
