# Supplementary material for: Multi-Variant Pathway Association Analysis Reveals the Importance of Genetic Determinants of Estrogen Metabolism in Breast and Endometrial Cancer Susceptibility
Source: PLoS Genet. 2010 Jul 1;6(7):e1001012. doi: 10.1371/journal.pgen.1001012 (PMC2895650; doi:10.1371/journal.pgen.1001012)
Supplement: Table S4 — Twenty-five most significant SNPs for breast cancer in Swedish sample. (0.07 MB DOC) [file pgen.1001012.s004.doc]

**Table S4.** Twenty-five most significant SNPs for Breast Cancer in Swedish sample.

| **Gene** | **SNP** | **P-value^a^** | **Adjusted P-value^b^** | **OR (95% CI)^c^** | **Cases /Controls^d^** | **MAF^e^** |
| --- | --- | --- | --- | --- | --- | --- |
| CYP19A1 | rs7167936 | 3.37*10^-4^ | 0.08 | 0.829 (0.748, 0.919) | 1512/1485 | 0.463 |
| CYP19A1 | rs12050767 | 0.001 | 0.24 | 1.184 (1.068, 1.313) | 1458/1453 | 0.47 |
| CYP19A1 | rs4646 | 0.008 | – | 0.858 (0.767, 0.961) | 1511/1483 | 0.284 |
| UGT2B4 | rs2736520 | 0.014 | – | 0.841 (0.733, 0.966) | 1461/1464 | 0.165 |
| AKR1C4 | rs7085249 | 0.014 | – | 1.153 (1.029, 1.293) | 1513/1468 | 0.266 |
| AKR1C4 | rs12762017 | 0.015 | – | 0.827 (0.710, 0.964) | 1295/1496 | 0.137 |
| UGT2B11 | rs4371687 | 0.015 | – | 1.131 (1.024, 1.249) | 1508/1485 | 0.492 |
| CYP19A1 | hCV3060064 | 0.016 | – | 0.881 (0.795, 0.977) | 1506/1478 | 0.446 |
| HSD17B3 | rs8190479 | 0.017 | – | 0.785 (0.643, 0.958) | 1325/1491 | 0.077 |
| UGT2B11 | rs6837285 | 0.018 | – | 0.887 (0.804, 0.979) | 1505/1467 | 0.491 |
| CYP19A1 | hCV8234885 | 0.018 | – | 0.881 (0.793, 0.979) | 1392/1452 | 0.397 |
| AKR1C4 | rs4880716 | 0.018 | – | 1.147 (1.023, 1.287) | 1508/1474 | 0.263 |
| UGT1A1.9 | rs2741019 | 0.02 | – | 0.875 (0.781, 0.979) | 1510/1496 | 0.278 |
| CYP19A1 | rs8031463 | 0.021 | – | 0.751 (0.588, 0.958) | 1516/1500 | 0.045 |
| CYP1A1.2 | rs6495121 | 0.033 | – | 0.843 (0.720, 0.986) | 1466/1481 | 0.119 |
| CYP19A1 | rs934632 | 0.038 | – | 0.874 (0.770, 0.992) | 1526/1482 | 0.195 |
| HSD11B1 | rs11576775 | 0.046 | – | 0.876 (0.769, 0.998) | 1475/1437 | 0.187 |
| NQO1 | rs1075935 | 0.05 | – | 1.320 (0.998, 1.744) | 1496/1451 | 0.036 |
| HSD11B1 | rs846906 | 0.053 | – | 0.872 (0.759, 1.002) | 1517/1483 | 0.158 |
| NAT1 | rs6586714 | 0.059 | – | 0.858 (0.731, 1.006) | 1515/1492 | 0.115 |
| SULT2B1 | rs3815691 | 0.064 | – | 0.871 (0.753, 1.008) | 1525/1503 | 0.136 |
| UGT1A1.9 | rs11563250 | 0.069 | – | 1.144 (0.990, 1.322) | 1515/1492 | 0.143 |
| CYP1A1.2 | rs1799814 | 0.069 | – | 0.761 (0.567, 1.023) | 1526/1489 | 0.031 |
| SULT2B1 | rs279447 | 0.075 | – | 0.803 (0.630, 1.023) | 1538/1506 | 0.045 |
| UGT2B11 | rs11932983 | 0.078 | – | 1.139 (0.985, 1.316) | 1513/1469 | 0.138 |

a .P-value of association using CA trend-test (rounded to 3 decimals)

b. P-value adjusted by Bonferroni correction (n=239); "–", adjusted P-value >1

c. Odds ratio and corresponding 95% confidence interval

d. Number of Control and Cases

e. Minor Allele Frequencies in control
