# Supplementary material for: Multi-Variant Pathway Association Analysis Reveals the Importance of Genetic Determinants of Estrogen Metabolism in Breast and Endometrial Cancer Susceptibility
Source: PLoS Genet. 2010 Jul 1;6(7):e1001012. doi: 10.1371/journal.pgen.1001012 (PMC2895650; doi:10.1371/journal.pgen.1001012)
Supplement: Table S5 — Twenty-five most significant SNPs for endometrial cancer in Swedish sample. (0.07 MB DOC) [file pgen.1001012.s005.doc]

**Table S5**. Twenty-five most significant SNPs for Endometrial Cancer in Swedish sample.

| **Gene** | **SNP** | **P-value^a^** | **Adjusted P-value^b^** | **OR (95% CI)^c^** | **Cases /Controls^d^** | **MAF^e^** |
| --- | --- | --- | --- | --- | --- | --- |
| CYP19A1 | rs12595627 | 1.67*10^-4^ | 0.04 | 0.766 (0.666, 0.882) | 661/1502 | 0.318 |
| CYP19A1 | rs934632 | 0.001 | 0.24 | 0.761 (0.645, 0.897) | 697/1529 | 0.198 |
| CYP19A1 | rs4646 | 0.003 | 0.72 | 0.805 (0.698, 0.929) | 678/1531 | 0.287 |
| CYP19A1 | rs7167936 | 0.003 | 0.72 | 0.825 (0.727, 0.937) | 693/1534 | 0.471 |
| CYP19A1 | rs12050767 | 0.007 | – | 1.191 (1.048, 1.353) | 677/1501 | 0.462 |
| HSD17B4 | rs154632 | 0.015 | – | 1.184 (1.034, 1.355) | 678/1520 | 0.335 |
| UGT2B11 | rs6600903 | 0.017 | – | 1.165 (1.027, 1.320) | 692/1521 | 0.391 |
| CYP19A1 | rs8031463 | 0.02 | – | 1.379 (1.055, 1.804) | 693/1547 | 0.054 |
| NAT2 | rs2410556 | 0.023 | – | 1.249 (1.033, 1.510) | 639/1484 | 0.117 |
| SULT2B1 | rs279447 | 0.024 | – | 0.698 (0.507, 0.962) | 700/1553 | 0.047 |
| CYP19A1 | hCV8234885 | 0.024 | – | 0.860 (0.754, 0.981) | 666/1501 | 0.404 |
| HSD17B4 | rs439954 | 0.027 | – | 0.803 (0.659, 0.978) | 638/1505 | 0.138 |
| HSD3B1 | rs911245 | 0.027 | – | 1.165 (1.018, 1.334) | 672/1492 | 0.341 |
| HSD17B3 | rs2066485 | 0.031 | – | 1.224 (1.020, 1.469) | 688/1552 | 0.14 |
| HSD3B1 | rs10923844 | 0.032 | – | 1.162 (1.014, 1.333) | 672/1522 | 0.302 |
| HSD3B1 | rs2298029 | 0.034 | – | 1.156 (1.012, 1.322) | 692/1536 | 0.335 |
| CYP19A1 | hCV3060064 | 0.038 | – | 0.873 (0.767, 0.993) | 685/1520 | 0.454 |
| HSD17B2 | rs2966244 | 0.038 | – | 0.579 (0.336, 0.996) | 699/1550 | 0.018 |
| CYP19A1 | rs17523880 | 0.05 | – | 1.203 (1.001, 1.446) | 694/1540 | 0.131 |
| GSTP1 | rs656652 | 0.055 | – | 1.134 (0.997, 1.290) | 695/1540 | 0.486 |
| SRD5A2 | rs12470143 | 0.059 | – | 1.131 (0.995, 1.286) | 655/1463 | 0.473 |
| SULT2A1 | rs4802397 | 0.063 | – | 0.802 (0.634, 1.015) | 406/858 | 0.155 |
| COMT | rs12484658 | 0.065 | – | 1.259 (0.988, 1.603) | 691/1542 | 0.068 |
| UGT2B11 | rs2331627 | 0.068 | – | 1.160 (0.990, 1.359) | 678/1509 | 0.197 |
| UGT2B11 | rs7677996 | 0.074 | – | 1.129 (0.989, 1.289) | 665/1465 | 0.322 |

a .P-value of association using CA trend-test (rounded to 3 decimals)

b. P-value adjusted by Bonferroni correction (n=239); "–", adjusted P-value >1

c. Odds ratio and corresponding 95% confidence interval

d. Number of Control and Cases

e. Minor Allele Frequencies in control
