# Supplementary material for: Multi-Variant Pathway Association Analysis Reveals the Importance of Genetic Determinants of Estrogen Metabolism in Breast and Endometrial Cancer Susceptibility
Source: PLoS Genet. 2010 Jul 1;6(7):e1001012. doi: 10.1371/journal.pgen.1001012 (PMC2895650; doi:10.1371/journal.pgen.1001012)
Supplement: Table S6 — Twenty-five most significant SNPs for breast cancer in Finnish sample. (0.07 MB DOC) [file pgen.1001012.s006.doc]

**Table S6.** Twenty-five most significant SNPs for Breast Cancer in Finnish sample.

| **Gene** | **SNP** | **P-value^a^** | **Adjusted P-value^b^** | **OR (95% CI)^c^** | **Cases/Controls^d^** | **MAF^e^** |
| --- | --- | --- | --- | --- | --- | --- |
| UGT2B4 | rs1494798 | 0.000 | 0.009 | 1.247 (1.119, 1.389) | 2212/1261 | 0.271 |
| UGT2B4 | rs3815691 | 0.002 | 0.255 | 0.826 (0.731, 0.933) | 2160/1225 | 0.22 |
| HSD11B1 | rs11576775 | 0.003 | 0.401 | 1.204 (1.063, 1.364) | 2202/1273 | 0.179 |
| UGT2B4 | rs6600771 | 0.007 | 0.812 | 0.834 (0.73, 0.951) | 2227/1276 | 0.172 |
| SULT2B1 | rs1132054 | 0.008 | 0.973 | 1.143 (1.035, 1.262) | 2208/1256 | 0.406 |
| HSD11B2 | rs7206718 | 0.012 | _ | 0.876 (0.793, 0.968) | 2161/1238 | 0.46 |
| UGT2B4 | rs903445 | 0.012 | _ | 1.14 (1.03, 1.263) | 2212/1254 | 0.346 |
| SRD5A2 | rs2300697 | 0.016 | _ | 1.131 (1.024, 1.25) | 2212/1270 | 0.387 |
| SULT2B1 | rs3848542 | 0.016 | _ | 1.152 (1.028, 1.29) | 2225/1275 | 0.233 |
| HSD11B1 | rs846906 | 0.036 | _ | 1.148 (1.008, 1.308) | 2204/1274 | 0.162 |
| SRD5A2 | rs6749019 | 0.045 | _ | 1.106 (1.002, 1.22) | 2199/1268 | 0.434 |
| SULT2B1 | rs10426628 | 0.048 | _ | 0.901 (0.814, 0.998) | 2193/1258 | 0.372 |
| HSD11B2 | rs4360931 | 0.063 | _ | 1.243 (0.989, 1.563) | 2214/1277 | 0.044 |
| SULT2A1 | rs188440 | 0.064 | _ | 1.112 (0.992, 1.246) | 2216/1257 | 0.238 |
| SRD5A2 | rs12470143 | 0.074 | _ | 0.914 (0.829, 1.008) | 2202/1269 | 0.5 |
| AKR1C4 | rs12762017 | 0.081 | _ | 1.122 (0.987, 1.275) | 2199/1253 | 0.172 |
| HSD3B1 | rs4659175 | 0.081 | _ | 0.908 (0.815, 1.011) | 2211/1265 | 0.296 |
| SULT2A1 | rs4483956 | 0.091 | _ | 1.094 (0.985, 1.215) | 2209/1268 | 0.31 |
| UGT1A1-9 | rs6719561 | 0.094 | _ | 1.091 (0.985, 1.209) | 2217/1267 | 0.345 |
| AKR1C4 | rs1931679 | 0.095 | _ | 0.876 (0.749, 1.024) | 2219/1269 | 0.115 |
| CYP19A1 | rs3751592 | 0.101 | _ | 0.911 (0.817, 1.016) | 2216/1258 | 0.286 |
| SULT2A1 | rs2972612 | 0.104 | _ | 1.093 (0.98, 1.218) | 2221/1266 | 0.275 |
| HSD11B2 | rs11642680 | 0.116 | _ | 0.835 (0.667, 1.046) | 2214/1269 | 0.053 |
| SULT2B1 | rs2665577 | 0.144 | _ | 0.928 (0.84, 1.026) | 2207/1269 | 0.399 |
| SRD5A1 | rs531241 | 0.150 | _ | 1.074 (0.974, 1.185) | 2203/1270 | 0.435 |

a .P-value of association using CA trend-test (rounded to 3 decimals)

b. P-value adjusted by Bonferroni correction (n=118); "–", adjusted P-value >1

c. Odds ratio and corresponding 95% confidence interval

d. Number of Control and Cases

e. Minor Allele Frequencies in control
